# Phosphate Metabolic Inhibition Contributes to Irradiation-Induced Myelosuppression through Dampening Hematopoietic Stem Cell Survival

**DOI:** 10.3390/nu14163395

**Published:** 2022-08-18

**Authors:** Yiding Wu, Weinian Liao, Jun Chen, Chaonan Liu, Shuzhen Zhang, Kuan Yu, Xinmiao Wang, Mo Chen, Song Wang, Xinze Ran, Yongping Su, Tianmin Cheng, Junping Wang, Changhong Du

**Affiliations:** 1 College of Preventive Medicine, Army Medical University, Chongqing 400038, China; 2 State Key Laboratory of Trauma, Burns and Combined Injury, Chongqing Engineering Research Center for Nanomedicine, Institute of Combined Injury, Chongqing 400038, China

**Keywords:** inorganic phosphate, myelosuppression, hematopoietic stem cell, irradiation, apoptosis

## Abstract

Myelosuppression is a common and intractable side effect of cancer therapies including radiotherapy and chemotherapy, while the underlying mechanism remains incompletely understood. Here, using a mouse model of radiotherapy-induced myelosuppression, we show that inorganic phosphate (Pi) metabolism is acutely inhibited in hematopoietic stem cells (HSCs) during irradiation-induced myelosuppression, and closely correlated with the severity and prognosis of myelosuppression. Mechanistically, the acute Pi metabolic inhibition in HSCs results from extrinsic Pi loss in the bone marrow niche and the intrinsic transcriptional suppression of soluble carrier family 20 member 1 (SLC20A1)-mediated Pi uptake by p53. Meanwhile, Pi metabolic inhibition blunts irradiation-induced Akt hyperactivation in HSCs, thereby weakening its ability to counteract p53-mediated Pi metabolic inhibition and the apoptosis of HSCs and consequently contributing to myelosuppression progression. Conversely, the modulation of the Pi metabolism in HSCs via a high Pi diet or renal Klotho deficiency protects against irradiation-induced myelosuppression. These findings reveal that Pi metabolism and HSC survival are causally linked by the Akt/p53–SLC20A1 axis during myelosuppression and provide valuable insights into the pathogenesis and management of myelosuppression.

## 1. Introduction

Cancer is becoming the leading cause of premature death in many countries [1]. Radiotherapy and chemotherapy are commonly effective approaches for cancer treatment. However, the distressing side effects including myelosuppression and gastrointestinal toxicity often hinder their clinical application [2]. Although new anticancer drugs, such as targeted drugs, are being developed to avoid these side effects, myelosuppression remains frequently inescapable [3]. Especially, myelosuppression is always life-threatening and is a primary cause for the discontinuation of cancer therapies [2]. Unfortunately, the pathogenesis of myelosuppression remains incompletely understood and there is a substantial unmet clinical need for new therapeutic strategies for myelosuppression.

Myelosuppression is defined as a condition in which the hematopoietic activity of bone marrow (BM) is suppressed, resulting in pancytopenia that is characterized by fewer output of white blood cells (WBCs), red blood cells (RBCs), and platelets [4]. The extensive and long-lasting suppression of BM hematopoiesis after cancer therapies indicates that hematopoietic stem cells (HSCs), which are the primitive progenitor of all blood cells, are injured by ionizing radiation (IR) or anticancer drugs [5,6,7]. In fact, HSCs are sensitive to cytotoxic agents, and will rapidly undergo cell death especially apoptosis upon exposed to them [6,7]. Importantly, the manipulation of apoptotic signaling in HSCs have been proven beneficial for the management of myelosuppression [8,9,10].

HSCs reside in a specialized microenvironment termed niche in the BM. Generally, it is thought that the HSC niche is formed by diverse niche cells including HSC descendants and nonhematopoietic cells. These niche cells provide a vital source of regulatory signals to ensure hematopoietic homeostasis by controlling the self-renewal and differentiation of HSCs [11]. Recent studies have shown that HSC niche cells are perturbed post IR and contribute greatly to HSC apoptosis and the consequent myelosuppression, spotlighting on a pathogenic role of the HSC niche disturbance in myelosuppression [11,12,13]. Notably, comparing to the rare HSCs, the HSC niche is much easier to target and manipulate. Thus, the HSC niche may be a more appropriate therapeutic candidate for myelosuppression. However, the roles and mechanisms of niche components in HSC injury remain largely unknown.

Recently, emerging studies including our own have revealed the distinct roles of niche nutrients, such as glucose [14], amino acid [15], calcium [16], iron [17], and inorganic phosphate (Pi) [18], in HSC maintenance. Though underappreciated, the nutrient distribution in the HSC niche may be somewhat different from the systemic distribution, thereby guaranteeing and/or restricting the availability of metabolic components to HSCs [19,20]. As known, besides myelosuppression, IR also systemically cause metabolic disturbances of a wide range of nutrients [21]. Nevertheless, it remains undefined whether the nutrient metabolism of HSCs is also perturbed during myelosuppression and its causal relationship with HSC injury.

In this study, using a mouse model of radiotherapy-induced myelosuppression, we show that myelosuppression accompanies Pi loss in the BM niche and an acute Pi metabolic inhibition in HSCs. Molecularly, Pi metabolism and HSC survival are causally linked by the Akt/p53–SLC20A1 axis. Importantly, the modulation of the Pi metabolism profoundly affects the severity and prognosis of myelosuppression through regulating HSC survival.

## 2. Materials and Methods

### 2.1. Animals and Diets

C57BL/6J-*Slc20a1^em1Smoc^* mice and *Kl^+/−^* mice were obtained as previously reported [18]. C57BL/6-*Trp53^tm1^*/Bcgen mice were purchased from Biocytogen Pharmaceuticals (Beijing, China). Normal C57BL/6 mice were purchased from Beijing HFK Bioscience Co., Ltd. (Beijing, China). All mice used were male, background-matched, and age-matched (8–10 weeks of age). For the IR experiment, mice were exposed to a single dose of 5 Gy total body irradiation by using a ^60^Co γ-ray source. The dose rate was 92.8 to 95.5 cGy/min. A control diet (CD) containing 0.6% phosphate and 1.0% calcium, and an HPD containing 1.65% phosphate and 1.0% calcium were purchased from Beijing HFK Bioscience Co., Ltd. (Beijing, China). Mice were adapted to CD or HPD for 1 week before IR exposure.

### 2.2. In Vivo Treatments

For the recombinant mouse Klotho (R&D Systems, Minneapolis, MN, USA) treatment, mice were administrated with a dose of 10 μg/kg intraperitoneally every other day, immediately post IR. For the MK-2206 (MedChem Express, Monmouth Junction, NJ, USA) treatment, mice were administrated with a dose of 4 mg/kg intraperitoneally every other day, immediately post IR.

### 2.3. Hematological Parameter Test

Hematological parameter test was performed as previously reported [22]. Briefly, 20 μL of blood was collected from the tail vein and diluted in 1% ethylene diamine tetraacetic acid solution and then counted automatically by a Sysmex XT-2000i hematology analyzer (Sysmex Corporation, Kobe, Japan).

### 2.4. HSC Pool Analysis and Sorting

BM cells (BMCs) were flushed from femurs and tibias, and RBCs were lysed with a red cell lysis buffer (Tiangen Biotech Co., Ltd., Beijing, China). HSCs (CD34^−^CD135^−^Lineage^−^Sca-1^+^c-Kit^+^) were analyzed using monoclonal antibodies as indicated and a lineage cocktail including CD3, Mac-1, Gr-1, B220, and Ter-119 (all eBioscience, San Diego, CA, USA). The HSC pool was analyzed using a FACSverse (BD Biosciences, San Jose, CA, USA) flow cytometer. Data analysis was performed using FlowJo software (Treestar Inc., San Carlos, CA, USA). Lineage^−^Sca-1^+^c-Kit^+^ (LSK) cells were sorted using a FACSAriaII (BD Biosciences) flow cytometer.

### 2.5. Cytoplasmic Protein Expression Analysis

To detect cytoplasmic protein expression, BMCs were firstly stained with surface markers for HSCs and carefully washed. BMCs were then fixed with IC Fixation buffer (eBioscience) at room temperature for 30 min and subsequently permeabilized with a permeabilization buffer (eBioscience) in the presence of anti-SLC20A1 (Abcam, Cambridge, UK), anti-p-Akt^S473^ (eBioscience), or anti-PUMA (Thermo Fisher Scientific, Carlsbad, CA, USA) antibodies at room temperature for another 30 min and finally analyzed by a FACSverse flow cytometer.

### 2.6. Nuclear Protein Expression Analysis

To detect nuclear protein expression, BMCs were firstly stained with surface markers for HSCs and carefully washed. BMCs were then resuspended with 1 mL of Foxp3 fixation/permeabilization working solution (eBioscience) at room temperature for 30 min. Subsequently, the BMCs were permeabilized with a permeabilization buffer (eBioscience) in the presence of anti-p53 (Thermo Fisher Scientific) antibodies at room temperature for another 30 min. Finally, the BMCs were stained with fluorescent-dye conjugated secondary antibodies (Thermo Fisher Scientific) and analyzed by a FACSverse flow cytometer.

### 2.7. HSC Apoptosis Analysis

HSC apoptosis was analyzed using an Annexin V-FITC Apoptosis Detection Kit (eBioscience) according to the manufacturer’s instructions. Briefly, BMCs were firstly stained with surface markers for HSCs and carefully washed. BMCs were then resuspended in 1 mL of 1× binding buffer. Then, annexin V-FITC antibody was added and incubated at room temperature for 10 min. After washing with 1× binding buffer for twice, a 7-amino-actinomycin D (7-AAD) staining solution (eBioscience) was added and immediately analyzed by a FACSverse flow cytometer. Apoptotic cells were identified as annexin V-positive cells.

### 2.8. Mitochondrial Membrane Potential (MMP) Analysis

The MMP of HSCs was analyzed using a tetramethylrhodamine ethyl ester (TMRE) dye (Thermo Fisher Scientific) according to the manufacturer’s instructions. Briefly, BMCs were firstly stained with surface markers for HSCs and carefully washed. Then, cells were washed and suspended in prewarmed (37 °C) 1 mL of Flow Cytometry Staining Buffer (Thermo Fisher Scientific) with 100 nM TMRE, together with 50 μM verapamil (Sigma-Aldrich, St. Louis, MO, USA). After been incubated at 37 °C for 30 min, cells were washed twice and immediately analyzed by a FACSverse flow cytometer.

### 2.9. Pi Determination

To determine the serum’s Pi contents, mice were anesthetized, and their blood were collected through cardiac puncture. Serum was collected by centrifuging at 1000× *g* for 10 min at room temperature. The Pi contents in the BM niche was analyzed by collecting BM extracellular fluid (BMECF) as previously reported [18]. To determine the Pi contents in LSK cells, sorted LSK cells were rinsed with normal saline and then lysed in a RIPA buffer (Sigma-Aldrich, St. Louis, MO, USA) supplemented with Pierce™ Phosphatase Inhibitor Mini Tablets and Pierce™ EDTA-free Protease Inhibitor Tablets (all Thermo Fisher Scientific) to inhibit the phosphatases and proteases. Then, samples were centrifuged at 14,000× *g* for 10 min and the supernatants were collected. The Pi contents in the serum, BM niche and LSK cells were assayed using a Phosphate Colorimetric Kit (Sigma-Aldrich) according to the manufacturer’s instructions. To avoid the interference of cell number, the total protein contents in sorted LSK cells were determined by a BCA protein concentration determination kit (Thermo Fisher Scientific) and used as a surrogate of total cell numbers. The Pi contents per LSK cell were shown in this study after normalized by their protein contents.

### 2.10. Gene Set Enrichment Analysis (GSEA)

RNA-sequencing (RNA-seq) data of HSCs from mice fed with an HPD for 3 months were acquired from Sequence Read Archive (accession number: PRJNA695319) [18]. A GSEA was performed using GSEA v4.2.3 (Broad Institute, Cambridge, MA, USA) based on Molecular Signatures Database v7.5.1 (http://software.broadinstitute.org/gsea/msigdb (accessed on 17 August 2022)).

### 2.11. Quantitative Polymerase Chain Reaction (qPCR)

To measure mRNA expression levels, RNA from sorted LSK cells was extracted using a RNeasy^®^ Micro Kit (QIAGEN, Hilden, Germany) according to the manufacturer’s instructions. The mRNA expression levels of *Slc20a1* were examined by qPCR as previously reported [18]. Data were normalized relative to hypoxanthine-guanine phosophoribosyltransferase (*Hprt*). Primer sequences were as follows: Forward-*Slc20a1*, 5′-GCTTCCGATTTCTGGGACCC-3′; Reverse-*Slc20a1*, 5′-CAGTTCAGACCACTTGACACC-3′. Forward-*Hprt*, 5′-TCAGTCAACGGGGGACATAAA-3′; Reverse-*Hprt*, 5′-GGGGCTGTACTGCTTAACCAG-3′.

### 2.12. Transcription Factor Binding Profile Analysis

The transcription factor binding profile of the *Slc20a1* gene was analyzed using two online databases, JASPAR (https://jaspar.genereg.net/ (accessed on 17 August 2022)) and AnimalTFDB 3.0 (http://bioinfo.life.hust.edu.cn/AnimalTFDB/#!/ (accessed on 17 August 2022)), by querying the murine *Slc20a1* promoter region 3000 bp upstream of the transcription start site, which was designated as +1. The putative binding sites of p53 that were predicted by both databases were selected for further verification.

### 2.13. Chromatin Immunoprecipitation (ChIP)

ChIP was performed as we previously reported according to the manufacturer’s instructions (Thermo Fisher Scientific) [22]. Briefly, hematopoietic stem and progenitor cells (HSPCs, c-Kit^+^ cells) were isolated using an EasySep™ Mouse CD117 (c-Kit) Positive Selection Kit (StemCell Technologies, Vancouver, BC, Canada) and were incubated with 1% formaldehyde for crosslinking. Then, HSPCs were lysed by sonicating on ice with several pulses. The cross-linked chromatin was subsequently coprecipitated with anti-p53 antibody (Cell Signaling Technology, Danvers, MA, USA) overnight. Finally, the harvested chromatin was purified for qPCR detection. The primers for ChIP are listed as follows: Forward-ChIP, 5′-GCGAGGTACAGATCGGGTC-3′, Reverse-ChIP, 5′-CACCAAGGTCGTTCCGAGTT-3′.

### 2.14. Statistical Analysis

A statistical analysis was performed using Prism v9.3.1 (GraphPad Software, La Jolla, CA, USA). All results are presented as mean ± standard deviation (SD). *n* represents the mouse number analyzed in each experiment, as described in the figure legends. Comparisons between two groups were determined by a two-tailed unpaired Student’s *t*-test. Three groups were compared by a one-way analysis of variance (ANOVA) followed by a Tukey–Kramer post hoc analysis. Kaplan–Meier curves and Log-rank test were used for survival analysis. *p* < 0.05 was considered statistically significant.

## 3. Results

### 3.1. Irradiation-Induced Myelosuppression Accompanies Pi Loss in BM Niche and Pi Metabolic Inhibition in HSCs

**Figure 1:**
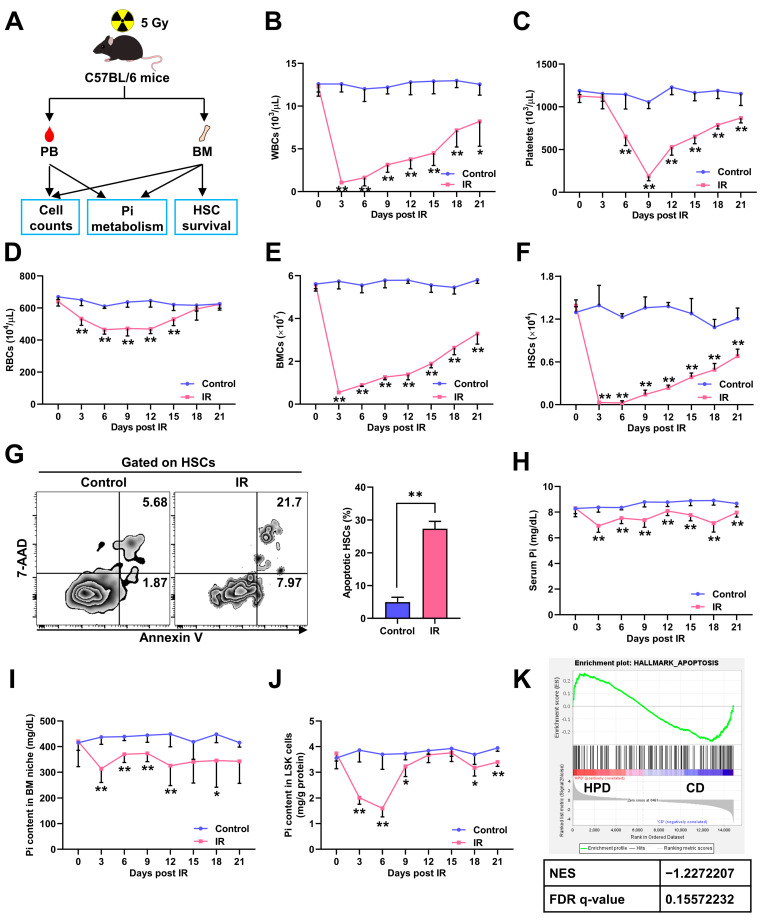
Irradiation-induced myelosuppression accompanies Pi loss in BM niche and Pi metabolic inhibition in HSCs. (**A**) Scheme for the mouse model of irradiation-induced myelosuppression and the analysis of cell counts, HSC survival, and Pi metabolism in peripheral blood (PB) and/or BM. (**B**–**D**) White blood cell (WBC), red blood cell (RBC), and platelet counts in PB of mice post IR (*n* = 6). (**E**) BMC counts post IR (*n* = 6). (**F**) HSC numbers in the BM of mice post IR (*n* = 6). (**G**) Representative flow cytometric analysis and quantification of apoptotic HSCs in the BM of mice at 3 dpi (*n* = 6). (**H**–**J**) Pi contents in serum, BM niche, and LSK cells of mice post IR (*n* = 6). (**K**) GSEA of apoptosis gene set in HSCs with enhanced Pi metabolism (PRJNA695319). Data are mean ± SD. * *p* < 0.05, ** *p* < 0.01. Two-tailed unpaired Student’s *t*-test.

Initially, mice were exposed to a sublethal dose (5 Gy) of IR to induce myelosuppression (Figure 1A), and dramatic pancytopenia (Figure 1B–D) and decrease in BMC numbers (Figure 1E) were observed. The pool size of BM HSCs also sharply decreased post IR, reaching a nadir by 6 days post IR (dpi) (Figure 1F). Accordingly, remarkable HSC apoptosis was detected at 3 dpi (Figure 1G). The BM hematopoietic activity slowly recovered from 3 dpi (Figure 1B–E), following the recovery of the HSC pool (Figure 1F), confirming that the HSC pool is a key factor that determines myelosuppression progression [6].

Recently, we reported that Pi metabolism regulated HSC maintenance at homeostasis [18]. Interestingly, during irradiation-induced myelosuppression, we noticed that the Pi contents in serum (Figure 1H) and BM niche (Figure 1I) were significantly declined. Meanwhile, the Pi contents in LSK cells, which are enriched with HSCs, were also sharply decreased, reaching a nadir by 6 dpi (Figure 1J). However, the Pi contents in LSK cells then rapidly recovered to normal levels thereafter (Figure 1J), though those in the serum and BM niche were continuously low (Figure 1H–I). Moreover, the decline of the Pi contents in LSK cells (Figure 1J) seemed more severe than that in the serum and BM niche (Figure 1H–I). These observations infer that the Pi metabolism of HSCs is more sensitive to IR and its inhibition may be only partly attributed to the Pi loss in the BM niche. Moreover, the change dynamics of the HSC pool (Figure 1F) and Pi contents in LSK cells (Figure 1J) were similar post IR, suggesting that acute Pi metabolic inhibition in HSCs may hamper HSC survival. Indeed, GSEA showed that enhanced Pi metabolism in HSCs was associated with a dampened apoptotic signaling (Figure 1K).

### 3.2. Pi Metabolic Inhibition Dampens HSC Survival Post IR

**Figure 2:**
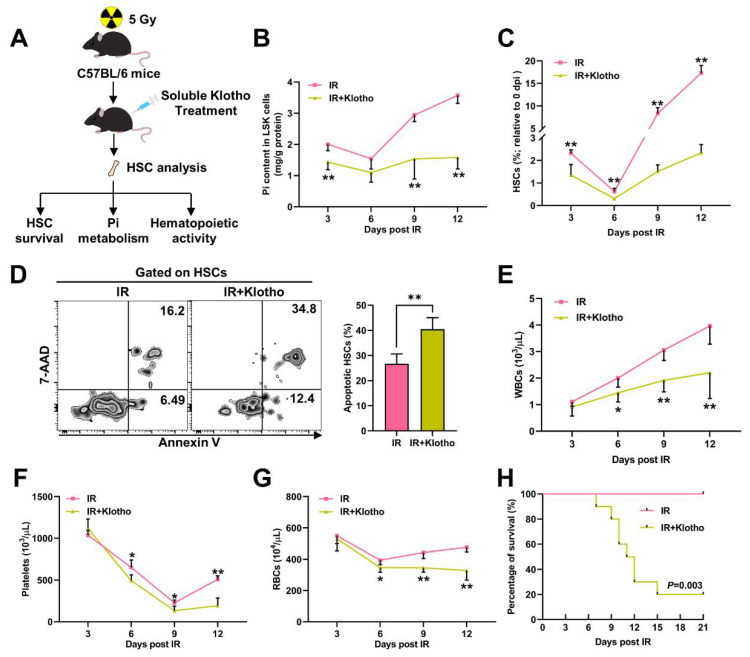
Inhibition of Pi metabolism by Klotho treatment dampens HSC survival post IR. (**A**) Scheme for Klotho treatment and HSC analysis. (**B**) Pi content in BM LSK cells of mice with or without Klotho treatment post IR (*n* = 6). (**C**) Relative HSC numbers in the BM of mice with or without Klotho treatment post IR compared to those at 0 dpi (*n* = 6). (**D**) Representative flow cytometric analysis and quantification of apoptotic HSCs in the BM of mice with or without Klotho treatment at 3 dpi (*n* = 6). (**E–G**) WBC, RBC, and platelet counts in PB of mice with or without Klotho treatment post IR (*n* = 6). (**H**) Survival rates of mice with or without Klotho treatment post IR (*n* = 10). Data are mean ± SD. * *p* < 0.05, ** *p* < 0.01. Two-tailed unpaired Student’s *t*-test. (**H**) Log-rank test.

We next intended to modulate the Pi metabolism of HSCs post IR. We firstly treated mice with Klotho, which is a circulating factor that inhibits Pi uptake of HSCs [18], immediately after IR. The Pi metabolism and survival of HSCs were monitored for 12 dpi (Figure 2A), a period when the inhibition of both is most severe. Surprisingly, the Klotho treatment evidently aggravated the Pi metabolic inhibition in LSK cells (Figure 2B). Meanwhile, irradiation-induced myelosuppression was exacerbated by the Klotho treatment, manifested by a smaller HSC pool (Figure 2C), more HSC apoptosis (Figure 2D), and more severe pancytopenia (Figure 2E–G). Consequently, the radiosensitivity of the mice was dramatically increased after the Klotho treatment (Figure 2H).

**Figure 3:**
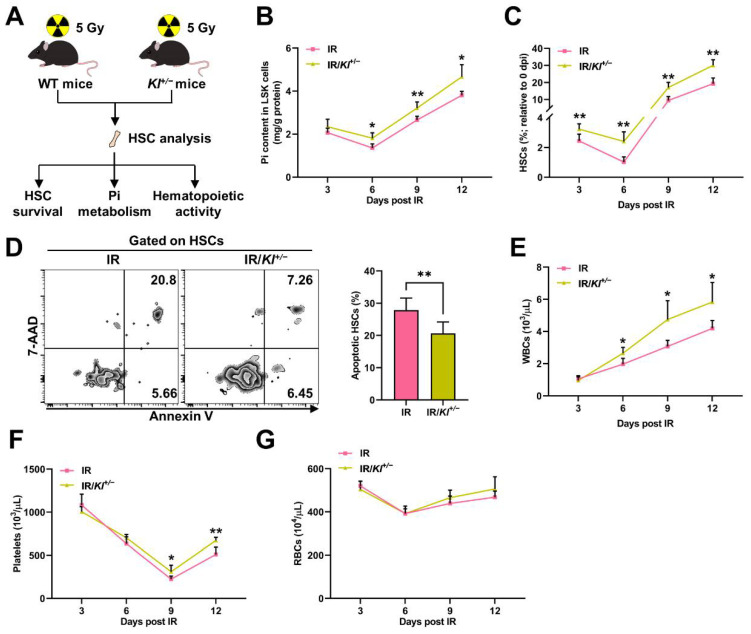
Enhancement of Pi metabolism by Klotho deficiency promotes HSC survival post IR. (**A**) Scheme for the creation of mouse model of irradiation-induced myelosuppression using *Kl^+/−^* mice and HSC analysis. (**B**) Pi content in BM LSK cells of WT and *Kl^+/−^* mice post IR (*n* = 6). (**C**) Relative HSC numbers in the BM of WT and *Kl^+/−^* mice post IR compared to those at 0 dpi (*n* = 6). (**D**) Representative flow cytometric analysis and quantification of apoptotic HSCs in the BM of WT and *Kl^+/−^* mice at 3 dpi (*n* = 6). (**E**–**G**) WBC, RBC, and platelet counts in PB of WT and *Kl^+/−^* mice post IR (*n* = 6). Data are mean ± SD. * *p* < 0.05, ** *p* < 0.01. Two-tailed unpaired Student’s *t*-test.

Conversely, Klotho haploinsufficiency significantly counteracted the IR-induced Pi metabolic inhibition in LSK cells (Figure 3B). More HSCs survived (Figure 3C) and less HSCs underwent apoptosis (Figure 3D) in the *Kl^+/−^* mice post IR, accompanied by significantly alleviated pancytopenia (Figure 3E–G). These results indicate that Pi metabolism affects HSC survival and is closely associated with the severity and prognosis of irradiation-induced myelosuppression.

### 3.3. SLC20A1 Is Indispensable for Pi Metabolism and Survival of HSCs Post IR

**Figure 4:**
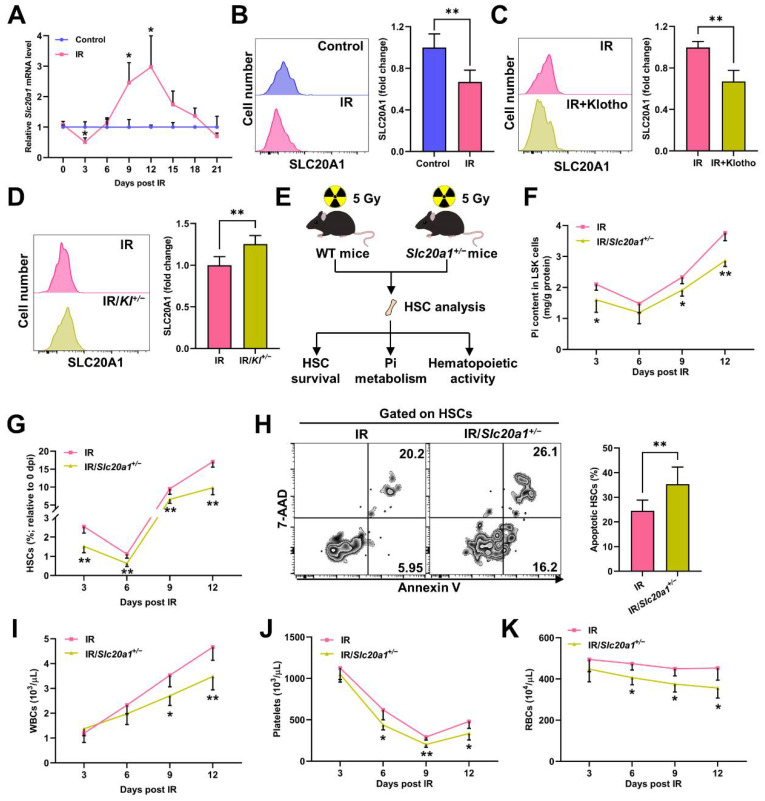
SLC20A1 is indispensable for Pi metabolism and survival of HSCs post IR. (**A**) Relative mRNA expression of *Slc20a1* in BM LSK cells of mice post IR (*n* = 3). (**B**) Representative flow cytometric analysis and fold change of mean fluorescence intensity (MFI) of SLC20A1 protein in BM HSCs of mice at 3 dpi (*n* = 6). (**C**) Fold change of SLC20A1 MFI in BM HSCs of mice with or without Klotho treatment at 3 dpi (*n* = 5). (**D**) Fold change of SLC20A1 MFI in BM HSCs of WT and *Kl^+/−^* mice at 3 dpi (*n* = 5). (**E**) Scheme for the creation of mouse model of irradiation-induced myelosuppression using *Slc20a1^+/−^* mice and HSC analysis. (**F**) Pi content in BM LSK cells of WT and *Slc20a1^+/−^* mice post IR (*n* = 6). (**G**) Relative HSC numbers in the BM of WT and *Slc20a1^+/−^* mice post IR compared to those at 0 dpi (*n* = 6). (**H**) Representative flow cytometric analysis and quantification of apoptotic HSCs in the BM of WT and *Slc20a1^+/−^* mice at 3 dpi (*n* = 6). (**I**–**K**) WBC, RBC, and platelet counts in PB of WT and *Slc20a1^+/−^* mice post IR (*n* = 6). Data are mean ± SD. * *p* < 0.05, ** *p* < 0.01. Two-tailed unpaired Student’s *t*-test.

Soluble carrier family 20 member 1 (SLC20A1) is the primary Pi transporter of HSCs and is positively regulated by Pi availability [18]. We found that the expression of Slc20a1 significantly downregulated at 3 dpi and thereafter began to upregulate (Figure 4A–B). Moreover, the Klotho treatment further downregulated (Figure 4C), while Klotho haploinsufficiency markedly upregulated SLC20A1 expression (Figure 4D) post IR. The change dynamics of the Pi contents (Figure 1J) and SLC20A1 expression (Figure 4A) were also similar in HSCs post IR, indicating that the intrinsic alteration of SLC20A1 expression might partially explain the fluctuation of the Pi contents in HSCs post IR as well. Indeed, SLC20A1 haploinsufficiency (*Slc20a1^+/−^*) significantly exacerbated the Pi metabolic inhibition in LSK cells post IR (Figure 4F). In accordance, the decline of the HSC pool size (Figure 4G), HSC apoptosis (Figure 4H), and pancytopenia (Figure 4I–K) were further aggravated in the *Slc20a1^+/−^* mice compared to their WT littermates post IR. Therefore, SLC20A1 is indispensable for the maintenance of Pi metabolism and survival of HSCs post IR.

### 3.4. Akt Counteracts Pi Metabolic Inhibition and Apoptosis of HSCs Post IR

**Figure 5:**
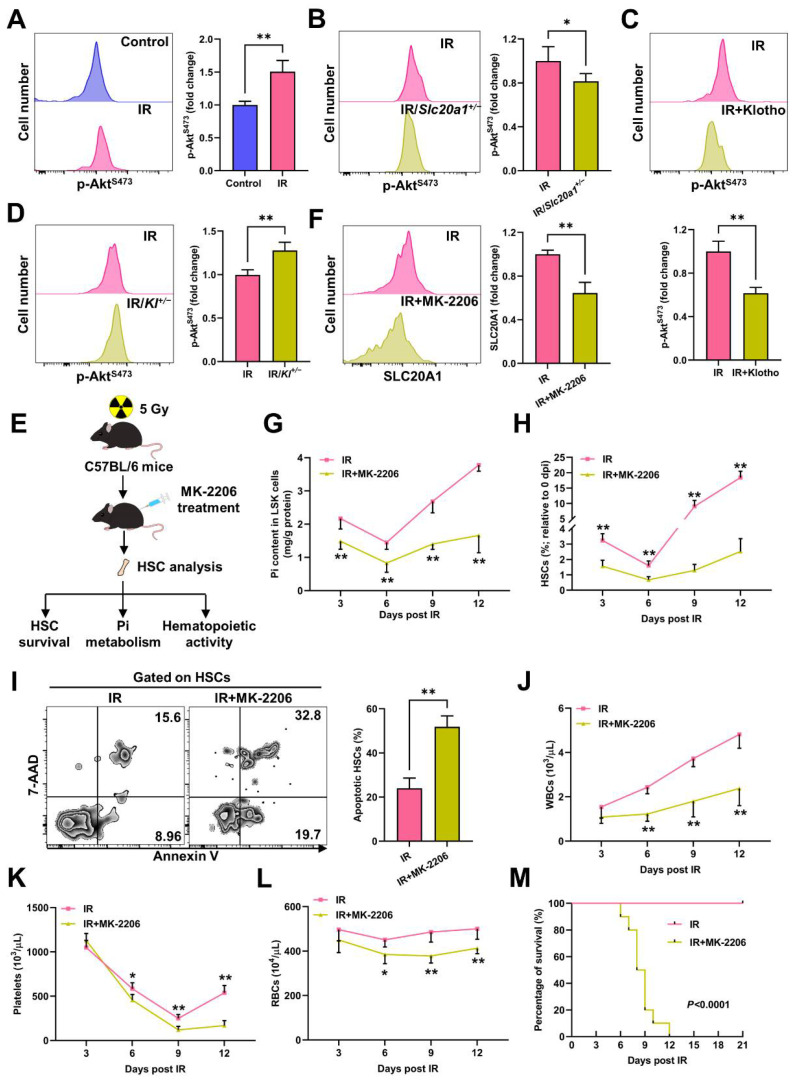
Akt counteracts Pi metabolic inhibition and apoptosis of HSCs post IR. (**A**) Representative flow cytometric analysis and fold change of p-Akt^S473^ MFI in BM HSCs of mice at 3 dpi (*n* = 5). (**B**) Representative flow cytometric analysis and fold change of p-Akt^S473^ MFI in BM HSCs of WT mice and *Slc20a1^+/−^* mice at 3 dpi (*n* = 5). (**C**) Representative flow cytometric analysis and fold change of p-Akt^S473^ MFI in BM HSCs of mice with or without Klotho treatment at 3 dpi (*n* = 5). (**D**) Representative flow cytometric analysis and fold change of p-Akt^S473^ MFI in BM HSCs of WT and *Kl^+/−^* mice at 3 dpi (*n* = 5). (**E**) Scheme for MK-2206 treatment and HSC analysis. (**F**) Representative flow cytometric analysis and fold change of SLC20A1 MFI in BM HSCs of mice with or without soluble MK-2206 treatment at 3 dpi (*n* = 5). (**G**) Pi content in BM LSK cells of mice with or without MK-2206 treatment post IR (*n* = 6). (**H**) Relative HSC numbers in the BM of mice with or without MK-2206 treatment post IR compared to those at 0 dpi. (*n* = 6). (**I**) Representative flow cytometric analysis and quantification of apoptotic HSCs in the BM of mice with or without MK-2206 treatment at 3 dpi (*n* = 6). (**J**–**L**) WBC, RBC, and platelet counts in PB of mice with or without MK-2206 treatment post IR (*n* = 6). (**M**) Survival rates of mice with or without MK-2206 treatment post IR (*n* = 10). Data are mean ± SD. * *p* < 0.05, ** *p* < 0.01. Two-tailed unpaired Student’s *t*-test. (**M**) Log-rank test.

Pi availability can be signaled to Akt, whose activation will in turn upregulate SLC20A1 to promote Pi uptake [18]. Surprisingly, inconsistent with the declined Pi contents and SLC20A1 expression in HSCs, we observed a remarkable Akt activation in HSCs at 3 dpi (Figure 5A). Meanwhile, Akt activation was blunted in the HSCs of the *Slc20a1^+/−^* mice (Figure 5B) and mice with the Klotho treatment (Figure 5C), but was augmented in the HSCs of the *Kl^+/−^* mice (Figure 5D). When Akt was pharmacologically inhibited by MK-2206, the IR-induced SLC20A1 downregulation in HSCs was exacerbated (Figure 5F), accompanied by an aggravated Pi metabolic inhibition in LSK cells (Figure 5G). These results indicate that the “SLC20A1–Akt–SLC20A1” circuit was still operational during irradiation-induced myelosuppression. Thus, the SLC20A1 downregulation in the context of Akt activation informs that another factor dominates SLC20A1 downregulation post IR. In addition, we observed that the HSC pool was much smaller (Figure 5H) and more HSCs underwent apoptosis post IR (Figure 5I) in the mice with the MK-2206 treatment, resulting in more severe pancytopenia (Figure 5J–L) and enhanced radiosensitivity (Figure 5M). Thus, Pi metabolic inhibition blunts Akt activation, rendering HSCs more susceptible to IR-induced apoptosis, whereas IR induces Akt activation to counteract the Pi metabolic inhibition and apoptosis of HSCs post IR.

### 3.5. p53 Promotes Pi Metabolic Inhibition and Apoptosis of HSCs Post IR

**Figure 6:**
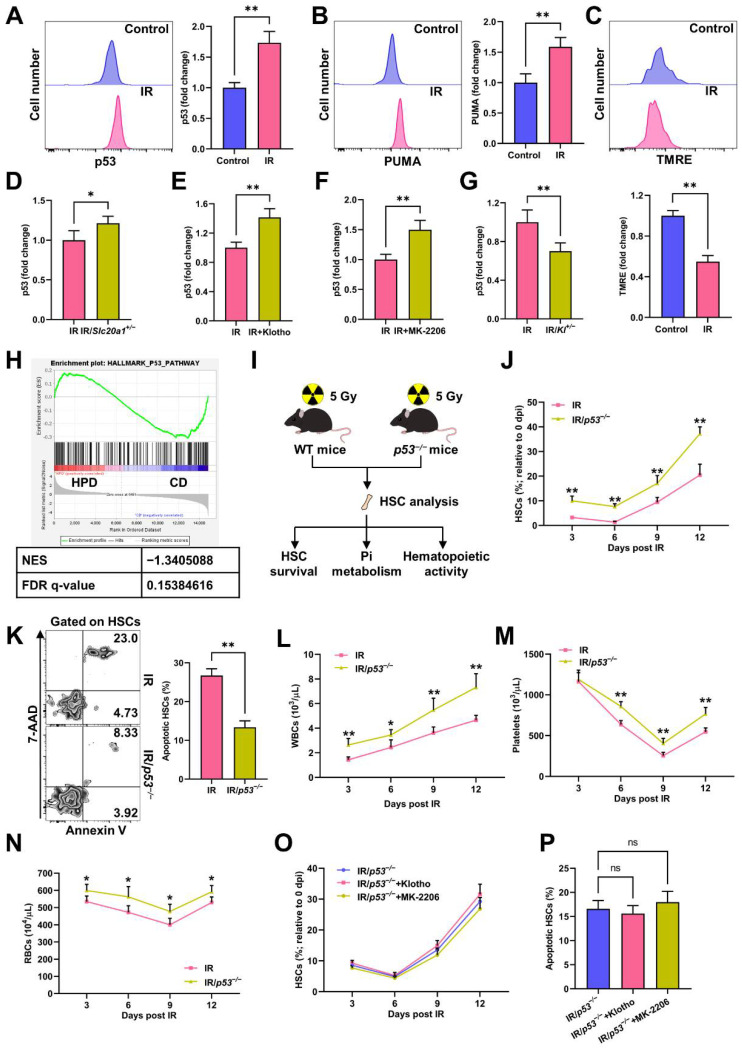
p53 promotes Pi metabolic inhibition and apoptosis of HSCs induced by IR. (**A**) Representative flow cytometric analysis and fold change of p53 MFI in BM HSCs of mice at 3 dpi (*n* = 5). (**B**) Representative flow cytometric analysis and fold change of PUMA MFI in BM HSCs of mice at 3 dpi (*n* = 5). (**C**) Representative flow cytometric analysis of MMP by TMRE staining and fold change of TMRE MFI in BM HSCs of mice at 3 dpi (*n* = 5). (**D**) Fold change of p53 MFI in BM HSCs of WT mice and *Slc20a1^+/−^* mice at 3 dpi (*n* = 5). (**E**) Fold change of p53 MFI in BM HSCs of mice with or without Klotho treatment at 3 dpi (*n* = 5). (**F**) Fold change of p53 MFI in BM HSCs of mice with or without MK-2206 treatment at 3 dpi (*n* = 5). (**G**) Fold change of p53 MFI in BM HSCs of WT and *Kl^+/−^* mice at 3 dpi (*n* = 5). (**H**) GSEA of p53 pathway gene set in HSCs with enhanced Pi metabolism (PRJNA695319). (**I**) Scheme for the creation of mouse model of irradiation-induced myelosuppression using *p53*^−/−^ mice and HSC analysis. (**J**) Relative HSC numbers in the BM of WT and *p53*^−/−^mice post IR compared to those at 0 dpi (*n* = 6). (**K**) Representative flow cytometric analysis and quantification of apoptotic HSCs in the BM of WT and *p53*^−/−^ mice at 3 dpi (*n* = 6). (**L**–**N**) WBC, RBC, and platelet counts in PB of WT and *p53*^−/−^ mice post IR (*n* = 6). (**O**) Relative HSC numbers in the BM of *p53*^−/−^ mice with or without Klotho or MK-2206 treatment post IR compared to those at 0 dpi (*n* = 5). (**P**) Frequency of apoptotic HSCs in the BM of *p53*^−/−^ mice with or without Klotho or MK-2206 treatment at 3 dpi (*n* = 5). Data are mean ± SD. ns = not significant. * *p* < 0.05, ** *p* < 0.01. Two-tailed unpaired Student’s *t*-test. (**O**,**P**) One-way ANOVA.

p53 and the downstream p53 upregulated modulator of apoptosis (PUMA)-mediated mitochondrial apoptosis signaling dominate cell apoptosis post IR [6]. Consistently, IR caused dramatic upregulation of p53 (Figure 6A) and PUMA (Figure 6B), as well as collapse of MMP (Figure 6C) in HSCs at 3 dpi. Of note, p53 activation was augmented in the HSCs of the *Slc20a1^+/−^* mice (Figure 6D) and mice with the Klotho treatment (Figure 6E) or MK-2206 treatment (Figure 6F), but was repressed in the HSCs of the *Kl^+/−^* mice (Figure 6G). Meanwhile, the GSEA showed that enhanced Pi metabolism in HSCs was associated with a dampened p53 signaling (Figure 6H). These data indicate that Pi metabolic inhibition is tightly linked with p53 activation in HSCs post IR. We then used *p53^−/−^* mice to determine the causal relationship between Pi metabolic inhibition and HSC apoptosis (Figure 6I). At homeostasis, the *p53^−^^/−^* mice exhibit a larger HSC pool than WT mice as well as comparable HSC apoptosis and blood cell counts (Figure S1). After IR exposure, the extents of HSC pool shrinking (Figure 6J), HSC apoptosis (Figure 6K), and pancytopenia (Figure 6L–N) were all less severe in the *p53^−/−^* mice comparing to those in their WT littermates, indicating that the *p53^−/−^* mice were more resistant to irradiation-induced myelosuppression. Meanwhile, the Klotho or MK-2206 treatments failed to exacerbate HSC apoptosis in the *p53^−/−^* mice (Figure 6O–P). Given that Akt is well identified as a suppressor of p53 signaling [23], these data indicate that Pi metabolic inhibition promotes p53-mediated HSC apoptosis via blunting Akt activation post IR.

**Figure 7:**
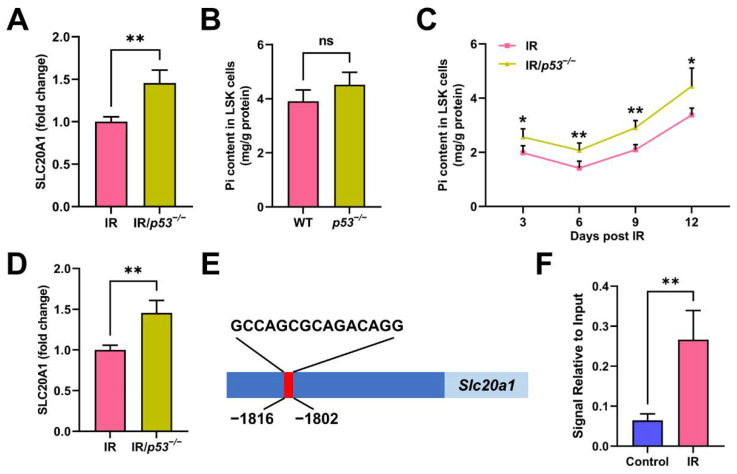
p53 transcriptionally restrains SLC20A1-mediated Pi uptake of HSCs. (**A**) Fold change of SLC20A1 MFI in BM HSCs of WT and *p53*^−/−^ mice without IR exposure (*n* = 5). (**B**) Pi content in BM LSK cells of WT and *p53*^−/−^ mice without IR exposure (*n* = 6). (**C**) Pi content in BM LSK cells of WT and *p53*^−/−^ mice post IR (*n* = 6). (**D**) Fold change of SLC20A1 MFI in BM HSCs of WT and *p53*^−/−^ mice at 3 dpi (*n* = 5). (**E**) The putative p53 binding site in the promoter of *Slc20a1* gene as predicted by both JASPAR and AnimalTFDB 3.0. (**F**) ChIP assay of p53 binding to the *Slc20a1* promoter sequence post IR (*n* = 3). Data are mean ± SD. ns = not significant. * *p* < 0.05, ** *p* < 0.01. Two-tailed unpaired Student’s *t*-test.

Interestingly, we also observed that SLC20A1 expression was significantly higher in the HSCs of the *p53^−/−^* mice than in that of the WT mice (Figure 7A), while the Pi contents in LSK cells were only moderately increased (Figure 7B). Meanwhile, the Pi metabolic inhibition in HSCs was remarkably alleviated in the *p53^−/−^* mice post IR (Figure 7C), accompanied by a higher SLC20A1 expression in HSCs (Figure 7D). These suggest that p53 may be a negative regulator of SLC20A1 expression and dominate SLC20A1 downregulation at 3 dpi. To test this hypothesis, we firstly analyzed the transcription factor binding profile of murine *Slc20a1* gene and found a putative p53 binding site in the promoter region (Figure 7E). Using ChIP, we verified that p53 indeed bound to the putative site in the promoter of the *Slc20a1* gene (Figure 7F). Moreover, the binding was further enhanced in HSPCs post IR (Figure 7F). These data demonstrate that p53 contributes to the IR-induced Pi metabolic inhibition in HSCs through transcriptionally repressing SLC20A1 expression.

### 3.6. Pi Supplementation Promotes HSC Survival and Protects against Irradiation-Induced Myelosuppression

**Figure 8:**
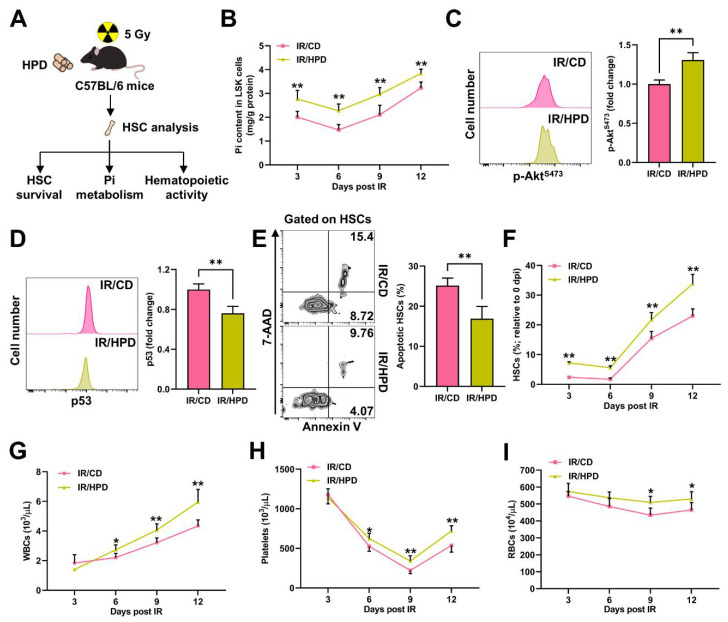
Pi supplementation promotes HSC survival and protects against irradiation-induced myelosuppression. (**A**) Scheme for HPD treatment and HSC analysis. (**B**) Pi content in BM LSK cells of mice with CD or HPD post IR (*n* = 6). (**C**) Representative flow cytometric analysis and fold change of p-Akt^S473^ MFI in BM HSCs of mice with CD or HPD at 3 dpi (*n* = 5). (**D**) Representative flow cytometric analysis and fold change of p53 MFI in BM HSCs of mice with CD or HPD at 3 dpi (*n* = 5). (**E**) Representative flow cytometric analysis and quantification of apoptotic HSCs in the BM of mice with CD or HPD at 3 dpi (*n* = 6). (**F**) Relative HSC numbers in the BM of mice with CD or HPD post IR compared to those at 0 dpi (*n* = 6). (**G**–**I**) WBC, RBC, and platelet counts in PB of mice with CD or HPD post IR (*n* = 6). Data are mean ± SD. * *p* < 0.05, ** *p* < 0.01. Two-tailed unpaired Student’s *t*-test.

Finally, we investigated whether correcting Pi metabolic inhibition could alleviate irradiation-induced myelosuppression. To achieve this, mice were adapted to an HPD for one week before IR and kept feeding an HPD post IR (Figure 8A). Comparing to mice with a CD, the HPD significantly alleviated the Pi metabolic inhibition in LSK cells (Figure 8B). Meanwhile, Akt activation was significantly enhanced (Figure 8C), and p53 activation was significantly blunted in the HSCs (Figure 8D) of the HPD mice post IR, resulting in decreased HSC apoptosis (Figure 8E), an increased HSC pool size (Figure 8F), and less severe myelosuppression (Figure 8G–I). Thus, the alleviation of Pi metabolic inhibition by Pi supplementation protects against irradiation-induced myelosuppression through promoting HSC survival.

## 4. Discussion

Emerging studies have shown that the perturbation of the HSC niche contributes substantially to the long course of myelosuppression and have identified that HSC niche cells, such as endothelial cells [24,25,26,27] and adipocytes [28], can be targeted to promote hematopoietic regeneration. Recent studies have also identified niche nutrients as distinct regulators of HSC homeostasis [16,20], while its implications in myelosuppression are largely unknown.

The BM niche has a relatively higher Pi concentration due to its location inside the bones, which are the Pi reservoir in the body. Since radiation injury always accompanies a severe decline in bone volume [29], it seems that the Pi homeostasis in the BM niche may be perturbed. As expected, the Pi contents in the BM niche kept low during irradiation-induced myelosuppression, accompanied by a sharp decline of the Pi contents in HSCs. Meanwhile, the change dynamic of the Pi contents in HSCs resembled that of the HSC pool post IR, pointing to a close interplay between Pi metabolism and HSC survival. Indeed, the aggravation of Pi metabolic inhibition by the soluble Klotho supplementation exacerbated HSC apoptosis, while the alleviation of Pi metabolic inhibition by Klotho haploinsufficiency prevented HSC apoptosis post IR. These findings demonstrate a distinct role of the Pi metabolism in HSC survival. More importantly, Pi metabolic inhibition is much easier to improve than the intrinsic HSC damage and niche cell dysfunction, and thus may hold great promise in the management of myelosuppression. Notably, in addition to inhibiting the Pi metabolism in HSCs, soluble Klotho is well known to act as an endogenous inhibitor of insulin-like growth factor 1 (IGF1) and Wnt [30,31], both of which have been reported to promote HSC survival during irradiation-induced myelosuppression [32,33]. Therefore, the inhibition of IGF1 and Wnt signalings together with the inhibition of Pi metabolism by soluble Klotho may synergistically exacerbate irradiation-induced HSC apoptosis.

As reported, Mk/myeloid regeneration dominates the early hematopoietic regeneration (at least by 21 dpi) during irradiation-induced myelosuppression [34]. In this study, we also observed a rapid recovery of Pi contents in HSCs following hematopoietic regeneration, inferring that Pi recovery may be a prerequisite for hematopoietic regeneration, especially for Mk/myeloid regeneration. Meanwhile, the expansion of the HSC pool and Mk/myeloid regeneration are accelerated when Pi metabolism is improved, but are blunted when Pi metabolism is further inhibited post IR, confirming that the Pi metabolism promotes the expansion and Mk/myeloid differentiation of HSCs as we previously reported [18]. Altogether, Pi metabolism may regulate apoptosis and the regeneration of HSCs, thereby contributing substantially to myelosuppression progression.

The Pi metabolism of HSCs is determined by both extracellular Pi availability and intracellular regulators. Akt is a pivotal positive regulator of Pi metabolism through upregulating SLC20A1 expression, and is activated in HSCs post IR. This may be attributed to DNA-damage-mediated activation of the gene mutated in ataxia telangiectasia (ATM) post IR [35]. Notably, SLC20A1 expression is inconsistent with Akt activity at 3 dpi, hinting that other factor may dominate the suppression of SLC20A1 expression and overwhelm the positive regulation of Akt activation. In this study, we reveal that p53 transcriptionally restrains SLC20A1 expression. Thus, the acute Pi metabolic inhibition in HSCs results from both extrinsic and intrinsic cues, involving the Pi loss in the BM niche as well as the intrinsic suppression of SLC20A1 by p53. However, unlike the continuous decline of the Pi contents in the BM niche, the Pi contents in HSCs rapidly recover following hematopoietic regeneration. This may be because of the deactivation of p53 and the resultant SLC20A1 upregulation that is induced by Akt activation during the regenerative phase. On the other hand, tumor cells, wherein p53 is always inactivated, are also associated with upregulated SLC20A1 expression and augmented Pi metabolism [36], further arguing the negative regulation of SLC20A1 expression by p53. Therefore, our findings may also aid the understanding of the progression and therapy resistance of cancers.

Pi homeostasis is tightly regulated by the balance of dietary Pi intake and renal Pi excretion, and by multiple hormones including parathyroid hormone, vitamin D, as well as fibroblast growth factor 23 (FGF23) and its coreceptor Klotho [37]. Among them, FGF23 is a potent phosphaturic hormone which can promote renal Pi excretion [38]. In animal models, the gastrointestinal system is sensitive while kidneys are resistant to IR [39]. Meanwhile, it is reported that FGF23 expression and secretion are dramatically increased post IR [40]. Therefore, the decreased intestinal Pi absorption and increased renal Pi excretion may collectively lead to the systemic Pi loss post IR. Consistent with this notion, an increased Pi intake and Klotho haploinsufficiency, which weakens the phosphaturic effect of FGF23 [41], protect against the Pi metabolic inhibition in HSCs post IR. In addition, hypophosphatemia is also frequently seen in emergency conditions such as HSC transplantation, inflammation, and infections [42,43], in which HSC survival also dominates their severity and prognosis [44,45]. It will be of great significance to investigate the mechanisms underlying the Pi metabolic disturbance in these conditions and their pathological roles.

## 5. Conclusions

**Figure 9:**
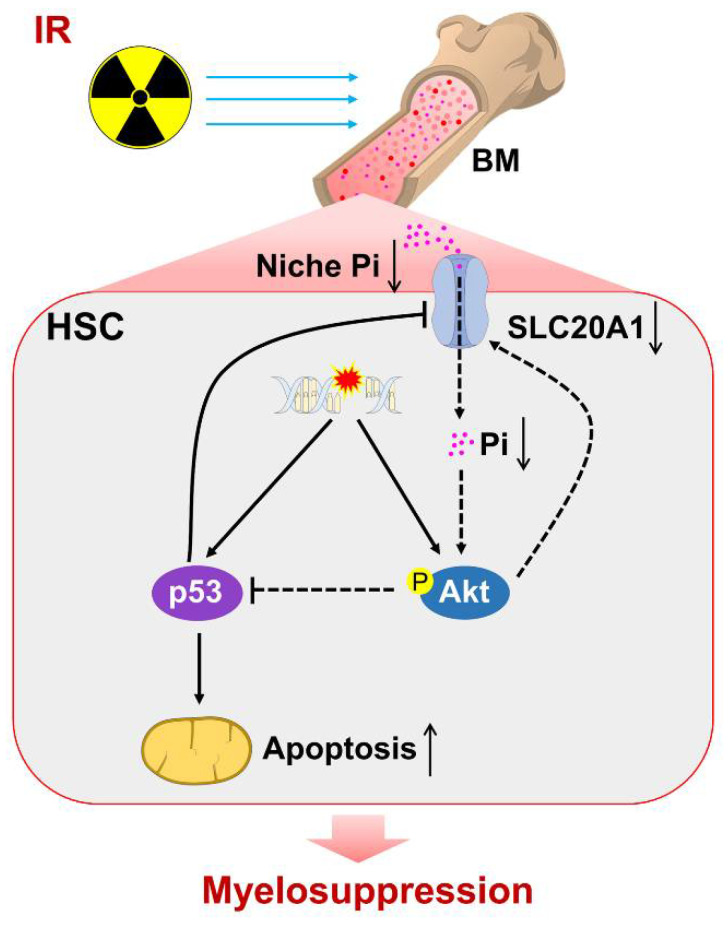
Scheme of Pi metabolic inhibition dampening HSC survival and contributing to irradiation-induced myelosuppression. ↑, activated. ↓, inhibited/decreased.

This study for the first time showed that the acute Pi metabolic inhibition in HSCs was a crucial pathologic feature of myelosuppression, as well as deeply uncovered the elaborate regulation of the Pi metabolism in HSCs during irradiation-induced myelosuppression as well as its pathogenic roles and therapeutic opportunities (Figure 9). These findings not only substantially extend our understanding of the regulatory role of niche nutrients in stem cell survival upon cytotoxic stress, but also provide valuable insights into the pathogenesis and management of myelosuppression.

## Data Availability

Data are contained within the article or Supplementary Materials. The RNA-seq datasets analyzed in this study are openly available in Sequence Read Archive (accession number: PRJNA695319).

## Supplementary Materials

The following supporting information can be downloaded at: https://www.mdpi.com/article/10.3390/nu14163395/s1, Figure S1: The steady-state hematopoiesis in *p53^−/−^* mice.
