# Supplementary material for: Phosphate Metabolic Inhibition Contributes to Irradiation-Induced Myelosuppression through Dampening Hematopoietic Stem Cell Survival
Source: Nutrients. 2022 Aug 18;14(16):3395. doi: 10.3390/nu14163395 (PMC9415467; doi:10.3390/nu14163395)
Supplement: Supplementary file 1 [file nutrients-14-03395-s001.zip › nutrients-1845698-supplementary.pdf]

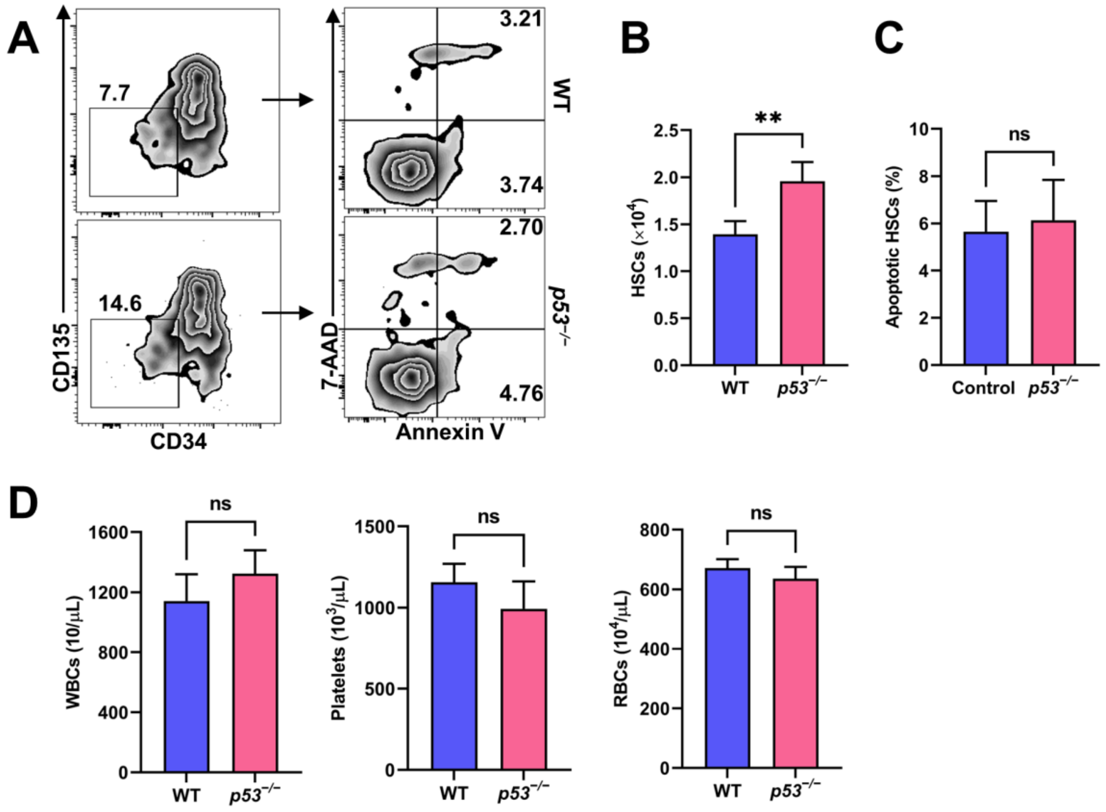

Figure S1. The steady-state hematopoiesis in *p53*$^{-/-}$ mice. (**A**) Representative flow cytometric analysis of pool size and apoptosis of BM HSCs of WT and *p53*$^{-/-}$ mice. (**B**) HSC numbers in the BM of WT and *p53*$^{-/-}$ mice (n = 6). (**C**) Frequency of apoptotic HSCs in the BM of WT and *p53*$^{-/-}$ mice (n = 6). (**D**) WBC, RBC, and platelet counts in PB of WT and *p53*$^{-/-}$ mice (n = 6). Data are mean ± SD. **p < 0.01. Two-tailed unpaired Student's *t*-test.
